# The German version of the helping alliance questionnaire: psychometric properties in patients with persistent depressive disorder

**DOI:** 10.1186/s12888-018-1697-8

**Published:** 2018-04-23

**Authors:** Hannah Sophie Eich, Levente Kriston, Elisabeth Schramm, Josef Bailer

**Affiliations:** 1 Central Institute of Mental Health, Department of Clinical Psychology, Medical Faculty Mannheim/Heidelberg University, J5, 68159 Mannheim, Heidelberg, Germany; 2 Department of Medical Psychology, University Medical Center Hamburg-Eppendorf, Martinistraße 52, 20246 Hamburg-Eppendorf, Hamburg, Germany; 3 Department of Psychiatry, Faculty of Medicine, University of Freiburg, Hauptstraße 5, 79104 Freiburg, Germany

**Keywords:** Helping alliance questionnaire (HAQ), Helping alliance, Therapeutic alliance, Psychometrics, Persistent depression

## Abstract

**Background:**

The Helping Alliance Questionnaire (HAQ) is a frequently used and highly relevant instrument to assess the therapeutic alliance. The questionnaire was translated into German by Bassler and colleagues (1995) and is available for patients (HAQ-P) and therapists (HAQ-T). Whereas the HAQ-P has been tested regarding psychometrics, the HAQ-T has not. This study aimed at further investigating the psychometric properties of both the HAQ-P and HAQ-T. We hypothesized that the instrument is reliable and shows factorial as well as convergent validity.

**Methods:**

Within the framework of a multisite, randomized-controlled clinical trial, comparing the efficacy of Cognitive Behavioral Analyses System of Psychotherapy (CBASP) and supportive psychotherapy (SP) in the treatment of early onset persistently depressed outpatients, the HAQ was filled out by patients (*n* = 255) and therapists (*n =* 81). 66.0% of patients were female; average age at randomization was 44.9 years (*SD* = 11.8). Several confirmatory factor analyses were conducted to test different structures for the HAQ. In addition, correlations between the HAQ and the Inventory of Interpersonal Problems (IIP) were calculated to test for convergent validity.

**Results:**

Goodness of fit indices for both a model with two different but strongly related factors named ‘relation to the patient/ therapist ’ and ‘satisfaction with therapeutic outcome’ and a second model with only one global helping alliance factor were comparable: Chi-Square-based indices rejected the models; RMSEA closely approached the threshold of good model fit, and CFI/ TLI and SRMR suggested that both models sufficiently fit the data. The internal consistency (Cronbach’s α) calculated for the different scales of the HAQ ranges between questionable to good. Finally, the HAQ scores were significantly related to some of the IIP scores.

**Conclusions:**

The German versions of the HAQ offer sufficient reliable instruments for the quick assessment of different facets of the therapeutic alliance. The HAQ global scores can be used as indicators for the global impression of the patients and therapists perception of the quality of the therapeutic alliance. However, the small correlations found between the IIP and the HAQ puts the question of external validity into perspective.

**Trial registration:**

This study analysed data from a RCT which was registered on ClinicalTrials.com (NCT00970437). First submitted on September 1, 2009.

## Background

The relationship between patient and therapist is one important factor in psychotherapy which predicts therapy outcome [1, 2]. One perspective on this relationship is the concept of alliance which was originally defined by Bordin [3] and “describes the degree to which the therapy dyad is engaged in collaborative, purposive work” [4]. Today, it is the most studied process variable in psychotherapy research [5]. Three internationally often used instruments to measure alliance are the Helping Alliance Questionnaire [6], the Working Alliance Inventory [7] and the California Psychotherapy Alliance Scales [8, 9]. The Working Alliance Inventory [7] is directly derived from Bordin’s theory of alliance [4]. It measures the agreement of patient and therapist on goals for and tasks in therapy as well as the affective bond between patient and therapist [7]. The California Psychotherapy Alliance Scales, which incorporates several perspectives on alliance, assesses the (i) patient’s commitment to therapy, (ii) the working capacity of the patient, (iii) the therapist’s understanding and involvement and (iv) the agreement of patient and therapist on goals and tasks [10]. The Helping Alliance Questionnaires (HAQ), of which one was investigated in this study, were developed by Alexander and Luborsky [11]. The first version of the HAQ was designed so that it encompasses two dimensions: HA1, i.e., the patient perceiving the therapist as helpful and supportive and HA2, i.e., working together towards common goals. Despite the two dimensions, authors themselves worked with the sum score of all items [12]. Later, Luborsky and colleagues developed a revised version of the HAQ, the HAq-II, in which the authors removed 6 items on early symptomatic improvement and added 14 items on the collaboration between patient and therapist, on how the patient perceived the therapist and on how the patients perceives the therapist’s feeling toward him or her [13]. The revised version however lacks sound psychometric testing and application in research [14]. Hence, while there is the HAq-II (for patients, therapists and observers), the HAQ is still widely used and recommended for research [15, 16].

The factor structure and psychometric properties of the HAQ have been investigated in six studies, which included in- and outpatient samples with heterogeneous diagnoses and receiving different forms of psychotherapy [9, 14, 17–20]. The studies generally confirmed the HAQ’s quality and its two factors: one related to the relationship and the other to outcome. Yet, the assignment of items to factors as well as the labelling of factors differed between studies. The authors attributed these discrepancies to differing statistical approaches (e.g., relying solely on exploratory factor analysis or allowing for correlated errors in confirmatory factor analysis), linguistic, cultural and scaling influences on responses, as well as differences in study setting such as study sample and treatment [9, 14, 17–20].

### German version of the HAQ

#### Factor structure

Only the first version of the HAQ was translated into German [18]. Like Alexander and Luborsky [11], the German authors found two factors [17, 18]. Yet, the assignment of items to factors and the number of items per factor differed to the originally proposed two dimensional structure by Alexander and Luborsky [11, 15, 16].

Two studies explicitly investigated the factor structure of the German version of the HAQ which is available in two versions – one for the patient (HAQ-P) and one for the therapist (HAQ-T). The earlier study [18] tested the HAQ-P in a sample of 239 psychodynamically treated inpatients with diverse diagnoses and found two factors, which they called ‘satisfaction with therapeutic outcome’ (items 2, 3, 4, 5, 11) and ‘relation to the therapist’ (items 1, 6, 7, 8, 9, 10). Item 2 and item 3 did not load clearly on either factor. Based on the items’ semantics, the authors assigned these items to the factor labelled ‘satisfaction with therapeutic outcome’. This assignment was later confirmed by test theoretical examination. Cronbach’s α of the global alliance scale was 0.89; the internal reliability of the subscales was similarly high (‘relation to the therapist’: Cronbach’s α = 0.89, ‘satisfaction with therapeutic outcome’: Cronbach’s α = 0.84). The intercorrelation between the factors was *r* = 0.43. Recently, a study by Nübling et al. [14] generally supported the two-factorial structure in a combined sample of three studies with in total 4626 in- and outpatients. Yet, items 1, 2 and 3 loaded inconsistently on the factors. Moreover, the fit indices of the confirmatory factor analyses suggested that the two items should be removed from the questionnaire. The authors however retained the two-dimensional structure of the HAQ-P including all 11 items for content-related reasons and because it is commonly used. The factors correlated between *r* = 0.45 and *r* = 0.76.

Hence, considering the equivocal findings with regards to the factor structure of the HAQ, the call for a sound psychometric foundation of the widely used HAQ-P [14] and its appropriateness to measure the helping alliance [21] remains open to discussion. Furthermore, psychometric examination of the German HAQ-T is entirely lacking. Therefore, this study aimed at adding to the already existing literature by testing the reliability and the factorial structure, of the German version of the HAQ-P and HAQ-T.

#### Validity

The German HAQ-P was found to have satisfactory convergent and discriminant validity as assessed by a number of variables which directly or indirectly measure therapy outcome and motivation for therapy [14, 17, 18]. We sought to expand the research on the validity of the HAQ by correlating the HAQ-P and HAQ-T with patients’ pre-treatment interpersonal problems. Patients with friendly-submissive behaviours facilitate a positive alliance with the therapist, whereas patients with hostile-dominant behaviours negatively impact alliance [22]. Also, McCullough [23] postulates that the hostile and hostile-submissive behaviours of persistently depressed patients impedes the interactions between patient and therapist. Hence, alliance scores should be negatively related to patients being overly hostile-dominant, hostile and hostile-submissive. Patients being friendly-submissive should be positively related to alliances scores.

#### Hypotheses

We hypothesised that the two theoretically proposed factors, i.e., ‘satisfaction with therapeutic outcome’ and ‘relation to the patient/ therapist’, would be confirmed and that the resulting subscales would be reliable and convergently valid.

## Methods

The hypotheses were tested with data from a randomized controlled trial (RCT) comparing the efficacy of the Cognitive Behavioural Analysis System of Psychotherapy (CBASP) to an active control group, i.e. supportive psychotherapy (SP). The RCT was registered on ClinicalTrials.com (NCT00970437). This prospective and observer-blind study was conducted at eight university centres throughout Germany. For details on the procedures, methodology, and outcome of the RCT see [24, 25].

### Participants

Patients between the age of 18 and 65 years, who had been diagnosed according to the Diagnostic and Statistical Manual of Mental Disorders (DSM-IV; American Psychiatric [26]) with an early onset (before the age of 21) diagnosis of chronic Major Depressive Disorder (MDD), current MDD superimposed on a pre-existing dysthymic disorder (“double depression”) or recurrent MDD with incomplete remission between episodes and scoring a minimum of 20 points on the 24-item Hamilton Rating Scale for Depression (HRSD) [27] were eligible to participate. Patients on antidepressant medication had the opportunity to discontinue it (at least two weeks of washout) before entering the trial. Unless any of the following exclusion criteria were met, patients were invited to take part in the study. Exclusion criteria were (i) acute suicidality, (ii) a history of psychotic symptoms, bipolar disorder, or organic brain disorder, (iii) a comorbid primary diagnosis of another axis I disorder, substance use disorder, (iv) antisocial, schizotypal, or borderline personality disorder, (v) severe cognitive impairment, (vi) non-response to CBASP and/or (SP) in an earlier trial, (vii) ongoing psycho−/pharmacotherapy, and (viii) a serious medical condition [25]. This sampling procedure resulted in 268 participants of whom 66% were female and who were on average 44.91 (*SD* = 11.82) years old (Table 1).

**Table 1:** Patient characteristics at baseline

Variable	Participants (*n* = 268)
Age, *M* (*SD*)	44.91 (11.82)
Gender, *n* (%)	
Male	91 (34.0)
Female	177 (66.0)
Age at onset, *M* (*SD*)	13.00 (4.41)
Marital status, *n* (%)	
Married; cohabiting	106 (39.6)
Single	117 (43.7)
Divorced, widowed	45 (16.8)
Educational level, *n* (%)	
≤ 11 years	96 (35.8)
≥ 12 years	172 (64.2)
Diagnosis^a^, *n* (%)	
Double Depression	119 (45.8)
Chronic Major Depression	82 (31.5)
Recurrent Major Depression without complete remission between episodes	59 (22.7)
Early trauma^a, b^, *n* (%)	194 (74.6)
CTQ global sum score^a^, *M* (*SD*)	52.82 (16.03)
Emotional abuse^a^, *M* (*SD*)	13.82 (5.57)
Physical abuse^a^, *M* (*SD*)	7.83 (4.21)
Sexual abuse^d^, *M* (*SD*)	6.52 (3.03)
Emotional neglect^e^, *M* (*SD*)	16.18 (5.02)
Physical neglect^a^, *M* (*SD*)	8.43 (3.17)
HRSD score^c^, *M* (*SD*)	27.07 (5.61)

*Notes.* ^a^ *n =* 260 (different to Schramm et al., 2017, we included belatedly collected (session 3) CTQ data for 4 participants for whom data was missing at baseline); ^b^ at least moderate to severe in 1 of 5 dimensions assessed with the Childhood Trauma Questionnaire; ^c^ *HRSD* Hamilton Rating Scale of Depression; ^d^ *n =* 258, ^e^ *n =* 259

### Treatments

Psychotherapy ran in parallel in both conditions (CBASP and SP) and included an acute therapy phase (20 weeks, 24 individual sessions) followed by eight continuation sessions over the next 28 weeks. CBASP is a highly structured intervention in which patients learn to recognize the effects of their behaviours on others, to actively deal with interpersonal problems and to strengthen self-efficacy by reaching their desired outcomes with other people. SP is a supportive, non-specific, client-centred approach to psychotherapy including elements of psychoeducation and facilitation of affect [28].

### Therapists

CBASP and SP were conducted by two groups of psychotherapists (*n* = 81), all of whom had either completed a three-year psychotherapy training or were in an advanced stage of training. In addition, therapists had been trained in a two-day workshop and had at least one practice day in either CBASP or SP. Before therapists began working with patients, they had to meet the criteria for mastery in CBASP or SP. During the therapy, all sessions were videotaped and supervision took place regularly.

### Measures

#### Demographic variables and early maltreatment

During the initial screening, sociodemographic variables, such as sex, age, nationality, marital status, education, occupation, and employment were recorded. Additionally, the Childhood Trauma Questionnaire (CTQ; [29]) was used to assess early traumatization in terms of emotional and physical abuse or neglect and sexual violence.

#### Diagnoses and depressive symptomatology

Before therapy commenced, clinical diagnoses had been derived from the Structured Clinical Interviews for DSM-IV (SCID I and II; [30, 31]) and severity of depressive symptoms had been quantified by the 24-item version of the HRSD.

#### Therapeutic alliance

Alliance was assessed at the beginning of therapy, i.e. after session 1. If the alliance questionnaire was not distributed and/ or was not returned after session 1, alliance was assessed after session 2 or 3. Both patient and therapist filled out the HAQ-P and HAQ-T [18], respectively. The 11 items of these self-report instruments take maximally 10 min to fill out. All items were rated on a 6-point Likert Scale from “strongly agree” (3 points) to “strongly disagree” (− 3 points).

#### Pretreatment interpersonal problems

We measured interpersonal problems by means of the German version of the Inventory of Interpersonal Problems (IIP-64; [32]). Its 64 items (5-point Likert scale) assess several aspects of social malfunctioning on 8 subscales which are correlated in the form of a circumplex: (i) domineering/ controlling, (ii) vindictive/ self-centred, (iii) cold/ distant, (iv) socially inhibited, (v) non-assertive, (vi) overly accommodating, (vii) self-sacrificing, (viii) intrusive/ needy. The subscales 1 through 4 describe problems with being too dominant, hostile, hostile-dominant, and hostile-submissive. The other subscales deal with problems concerning friendly submissiveness or friendly dominance. The questionnaire has been found to be a reliable and valid research instrument in English and German populations [32].

### Data analysis

To test the factor structure of the German HAQ for patients and therapists, we performed confirmatory factor analyses (CFA) by means of structural equation modelling with diagonally weighted least squares estimation (WLSMV). In the analyses items were modelled as ordinally scaled. Based on the recommendation to consider several tests when evaluating model fit [33], we included the following indices: Normed Chi-Square (Chi-Square Test of Model Fit divided by Degrees of Freedom), Root Mean Square Error of Approximation (RMSEA), Comparative Fit Index (CFI), Tucker Lewis Index (TLI) and the Standardized Root Mean Square Residual (SRMR). In order to interpret the fit indices we relied on the same cut-off values (Table 3) as Nübling et al. did in their study on the HAQ [14]. The models were investigated with regards to internal consistency and external validity. The internal consistency of the HAQ was assessed by use of Cronbach’s α. To externally validate the German HAQ as a measure to assess the therapeutic alliance between patient and therapist, we performed Pearson correlations (two-tailed) between HAQ and IIP (total score and subscale scores). All descriptive statistics, analyses on internal consistency and external validation were performed using IBM SPSS Statistics 24 [34]; lavaan for R [35] was used to perform the CFA.

## Results

The sample comprised of 268 patients (Table 1), 177 (66.0%) of whom were female, with an average age of 44.91 (*SD* = 11.82). Almost half of the sample was suffering from double depression (45.8%). The other patients had either chronic major depression (31.5%) or recurrent major depression without complete remission between episodes (22.7%). Patients reported a mean age of onset of 13 years (*SD* = 4.41). The average HRSD score at baseline was 27.07 (*SD* = 5.61). Most patients were single (43.7%) or married (39.6%), 16.8% were divorced or widowed. About one third (35.8%) of patients had been in formal education for at least 12 years. Over 70% reported early childhood maltreatment. Of the 255 patients, who returned the HAQ-P, 254 also filled out the IIP.

### Confirmatory factor analysis and internal consistency

To confirm the postulated two-dimensional factor structure of the HAQ [14, 17], we assumed a model with two latent factors in confirmatory factor analysis. This model was tested for both the patient and the therapist versions of the questionnaire. Structural equation modelling showed that 6 relationship items and 5 outcome items correlated significantly with the latent factors (Table 2) and that the two factors strongly correlated (HAQ-P: *r* = .83; HAQ-T: *r* = .88).

**Table 2:** Standardized factor loadings of items on factors for a two- and one-factorial model

	2 factors		1 factor
HAQ-P^a^	*Relation to the patient/ therapist *	*Satisfaction* *with therapeutic outcome*	*Helping alliance*
1: I believe that my therapist is helping me.	.91^c^		.88^c^
6: I feel I can depend on my therapist.	.75**		.74**
7: I feel the therapist understand me.	.78**		.77**
8: I feel the therapist wants me to achieve my goals.	.84**		.83**
9: I feel I am working together with the therapist in a joint effort.	.88**		.87**
10: I believe we have similar ideas about the nature of my problems.	.73**		.72**
2: I believe that the treatment is helping me.		.95^c^	.85**
3: I have obtained some new understanding.		.69**	.64**
4: I have been feeling better recently.		.65**	.62**
5: I can already see that I will eventually work out the problems I came to treatment for.		.76**	.70**
11: I feel now that I can understand myself and deal with myself on my own.		.22**	.21**
Correlation between factors	.83**		
*M* (*SD*)	1.56 (0.84)	−0.06 (1.15)	0.82 (0.88)
Cronbach’s *α*	.89	.75	.87
HAQ-T^b^			
1: I believe that I am helping my patient.	.77^c^		.76 ^c^
6: I feel that my patient relies on me.	.66**		.66**
7: I feel that my patient feels understood.	.87**		.86**
8: I feel my patient believes I am committed to the attainment of his/ her goals.	.91**		.90**
9: I feel that my patient is working together with me in a joint effort.	.79**		.78**
10: I believe my patient and I have similar ideas about the nature of his/ her problems.	.69**		.69**
2: I believe that the treatment is helping my patient.		.77^c^	.71**
3: I believe that my patient has obtained some new understanding.		.71**	.66**
4: I believe that my patient has recently been feeling better.		.32**	.30**
5: I believe my patient will eventually work out the problems he/she came to treatment with.		.57**	.53**
11: I feel now that my patient can understand him/ herself and can deal with him/ herself on his/ her own.		.28**	.26**
Correlation between factors	.88**		
*M* (*SD*)	0.91 (0.80)	−0.32 (0.83)	0.35 (0.72)
Cronbach’s *α*	.85	.63	.84

*Note*. ^a^ *n* = 255; ^b^ *n* = 260; ** *p* < .001; * *p* < .05; ^c^ reference item in the model; *HAQ-P* Helping Alliance Questionnaire for patients, *HAQ-T* Helping Alliance Questionnaire for Therapists

While the goodness of fit indices were mostly satisfactory for the HAQ-T (χ^2^ = 153.98, *df* = 43, *p* < .001, χ^2^/*df* = 3.58, RMSEA = .10, CFI = .98; TLI = .97, SRMR = .08), the indices were inconclusive for the HAQ-P (χ^2^ = 213.36, *df* = 43, *p* < .001, χ^2^/*df* = 4.96, RMSEA = .12, CFI = .98; TLI = .98, SRMR = .07) (Table 3). Cronbach’s α of the two scales ranged from .89 to .75 on the HAQ-P and from .85 to .63 on the HAQ-T.

**Table 3:** Goodness of fit indices for a two- and one-factorial model

Model	χ^2^	*df*	*p*	χ^2^/*df*	RMSEA	CFI/ TLI	SRMR
Two-factorial model							
HAQ-P	213.36	43	< .001	4.96	.12	.98/ .98	.07
HAQ-T	153.98	43	< .001	3.58	.10	.98/ .97	.08
One-factorial model							
HAQ-P	268.42	44	< .001	6.10	.14	.98/ .97	.09
HAQ-T	163.95	44	< .001	3.73	.10	.98/ .97	.08
Cut-off			> .05	< 3	≤.10	≥ .95/ ≥ .90	< .11

*Note*. *df* degrees of freedom, *RMSEA* root mean error of approximation, *CFI* comparative fit index, *TLI* Tucker Lewis index, *SRMR* standardized root mean square residual, *HAQ-P* Helping Alliance Questionnaire for Patients, *HAQ-T* Helping Alliance Questionnaire for Therapists

Because of the high correlation between the factors and cross-loadings between items and factors, we tested a competing one-factor model (Table 2). In the one-factor model all items load onto one latent global factor. This model had comparable or slightly inferior fit indices than the two-factor model: HAQ-P (χ^2^ = 268.42, *df* = 44, *p* < .001, χ^2^/*df* = 6.10, RMSEA = .14, CFI = .98; TLI = .97, SRMR = .09) and HAQ-T (χ^2^ = 163.95, *df* = 44, *p* < .001, χ^2^/*df* = 3.73, RMSEA = .10, CFI = .98; TLI = .97, SRMR = .08) (Table 3). Cronbach’s α of the global scale was .87 for the HAQ-P and .84 for the HAQ-T.

### External validity

Table 4 shows the correlations between the HAQ global alliance score and its subscales with the IIP total score and its subscales.

**Table 4:** Correlations between the HAQ and the IIP total score and its 8 subscales

		HAQ-P^b^				HAQ-T^c^		
		*Relation to the therapist*	*Satisfaction with therapeutic outcome*	*Helping alliance*		*Relation to the patient*	*Satisfaction with therapeutic outcome*	*Helping alliance*
IIP^a^	*M (SD)*	*r*	*r*	*r*		*r*	*r*	*r*
total score	14.92 (3.69)	−0.10	−0.15*	−0.14*		−0.05	− 0.05	−0.06
Domineering/ controlling	8.31 (5.10)	−0.14*	−0.10	− 0.13*		− 0.12	−0.12	− 0.13*
Vindictive/ self-centred	11.02 (5.19)	−0.17**	−0.18**	− 0.19**		− 0.11	−0.11	− 0.12
Cold/ distant	14.79 (6.04)	−0.21*	−0.21**	− 0.23**		− 0.08	−0.08	− 0.09
Socially inhibited	17.93 (6.71)	−0.16*	−0.23**	− 0.22**		− 0.11	−0.11	− 0.12*
Non-assertive	20.04 (6.68)	−0.03	−0.12	− 0.08		0.00	0.01	0.01
Overly accommodating	17.49 (5.83)	0.07	−0.00	0.03		0.10	0.10	0.11
Self-sacrificing	18.71 (5.29)	0.11	−0.01	0.06		0.08	0.08	0.09
Intrusive/ needy	11.10 (5.33)	0.05	−0.09	0.08		−0.01	−0.02	− 0.02

*Note*. ^a^ *n* = 254; ^b^ *n* = 255; ^c^ *n* = 260; ** *p* < .01, *p < .05; *HAQ-P* Helping Alliance Questionnaire for Patients, *HAQ-T* Helping Alliance Questionnaire for Therapists, *IIP* Inventory of Interpersonal Problems

#### HAQ-p

The IIP total score correlated significantly negatively with the HAQ-P global helping alliance score (*r* = − 0.14, *p* = .03) and with the subscale ‘satisfaction with therapeutic outcome’: *r* = − 0.15, *p* = .02). The HAQ-P global score also had a significant negative relationship with the following interpersonal problems: domineering/ controlling (*r* = − 0.13, *p* = .04), vindictive/ self-centred (*r* = − 0.19, *p* < .01), cold/ distant (*r* = − 0.23, *p* < .01), and socially inhibited (*r* = − 0.22, *p* < .01). Likewise, the subscale ‘relation to the therapist’ was negatively correlated with the same octants: domineering/ controlling (*r* = − 0.14, *p* = .03), vindictive/ self-centred (*r* = − 0.17, *p* = .01), cold/ distant (*r* = − 0.21, *p* < .01), and socially inhibited (*r* = − 0.16, *p* = .01). The HAQ-P subscale ‘satisfaction with therapeutic outcome’ was only related to the following IIP subscales: vindictive/ self-centred (*r* = − 0.18, *p* < .01), cold/ distant (*r* = − 0.21, *p* < .01), and socially inhibited (*r* = − 0.23, *p* < .01).

#### HAQ-t

For the HAQ-T, significant correlations were found between the helping alliance global scale and the octants domineering/ controlling (*r* = − 0.13, *p* = .03) and socially inhibited (*r* = − 0.12, *p* = .04).

## Discussion

We examined the psychometric properties of the German HAQ in a large sample of early-onset persistently depressed outpatients and their therapists. By means of structural equation modelling we sought to confirm the elsewhere [14] assumed two-factorial structure of the HAQ. Fit indices were heterogeneous: Chi-Square-based indices rejected the model; RMSEA closely approached the threshold of good model fit, and CFI/ TLI and SRMR suggested that this two-factorial model sufficiently fit the data. Because of the ambiguous fit indices, items cross loading on factors and the high correlation between the factors (HAQ-P: *r* = .83; HAQ-T: *r* = .88), we ran additional CFA on a competing one-factor model. The second analysis showed that a one-factorial model had a mostly comparable model fit. Standardized loadings of items on latent factors were generally high. Only item 11 (HAQ-P: “I feel now that I can understand myself and deal with myself on my own.”, HAQ-T: “I feel now that my patient can understand him/ herself and can deal with him/ herself on his/ her own.”) had consistent loadings of < .30 onto the factor ‘satisfaction with therapeutic outcome’ in the two-factorial model and on the global factor in the one-factorial model. This may be due to the fact that data was collected at the very beginning of treatment when agreeing to this item is unlikely. Other items can be agreed on earlier in the process of therapy. We expect that at a later point in the treatment, item 11 will load onto the factor labelled ‘satisfaction with therapeutic outcome‘, which it has been assigned to mainly for content-related reasons.

Generally, our findings mirror that of other studies on the psychometric properties of the HAQ: the HAQ, i.e., its global scale and its subscales are internally consistent. Like in other studies, we found very high correlation between the latent factors, which indicates how close, the dimensions ‘relation to the therapist/ patient’ and ‘satisfaction with therapeutic outcome’ are. While the inter-correlation between the latent factors parallels earlier findings, the magnitude of the herein reported correlation may have been overestimated due to using WLSMV estimation in a small sample [36].

Like in other studies which employed CFA to verify a theoretically proposed factor structure [37], our results failed to unambiguously confirm the HAQ scale(s). Our findings are partially in line with Nübling et al. [14]: they, too, found flaws in the two-factorial structure. In their analyses a two-factorial model without item 2 and 3 proved superior to the proposed model. Because of content related reasons and due to the dispersion of the two factor solution which includes all 11 items, the authors retained the unsatisfactory yet well-known model. No other German study has sought to confirm a one factor structure of the HAQ.

Previous research on the Dutch HAQ [20], too, compared the fit of a two- and a one-factor model of the HAQ and also found items cross-loading (two-factorial model) and correlated measurement errors in the models. Moreover, they had slightly inferior fit indices for the one- than for the two-factorial model, which is why they retained the two-factorial model.

In the literature the HAQ is used uni- and two-dimensionally. Our findings from CFA suggest that the model fit of a two- and one-factorial model is largely comparable. Hence, two models reasonably fit the data. Like in other herein cited studies, model fit was not persistently conclusive, but acceptable with regards to CFI, TLI, and SRMR. The finding that the fit indices are inconclusive is unfortunate but not surprising as they are differently susceptible to aspects of structural equation modelling (e.g., [38]).

It is known that most fit indices can be affected by sample size, but also by estimation method and other aspects [38, 39]. One study employed Maximum Likelihood (ML) and Generalized Least Squares (GLS) estimation and found that in comparison to other indices RMSEA and CFI were minimally influenced by sample size [38]. Regarding the herein employed diagonally weighted least squares estimation (WLSMV) method, there is, to our knowledge, no consensus as to how sample size affects the resulting fit indices. Rather, the WLSMV estimator has not been studied sufficiently yet [40]. Two studies investigating the effect of sample size on WLSMV estimation found that WLSMV performs equally well as ML across different sample sizes [40]. Another study [36] however found that in small samples (i.e., *n* = 200) models based on WLSMV tend to be over rejected by the Chi-Square Test. Therefore, the common assumption that Chi-Square based fit indices are lenient in small samples [38] may not hold true for our study. Rather, the unsatisfactory Chi-Square results in our study may be due to having relied on WLSMV estimation in a relatively small sample.

In light of the herein used methods and the results, looking at the global alliance scale or the subscales of the HAQ is both equally feasible. Therefore, the researcher or clinician will have to decide what approach better fits the purpose. Working with a two-factorial model holds the advantage of comparability: the subscales are well known and commonly used. Moreover, items on the scale ‘satisfaction with therapeutic outcome’ are confounded with therapy outcome [14]. Therefore, assessing alliance on both subscales allows a more fine graded disentanglement of process and outcome variables in psychotherapy research. On the other hand, assessing alliance on one global score is arguably very economic. What is more, relying on one global HAQ score is frequently done (e.g, [41]).

Previously, validity of the German HAQ-P had been established through symptom-, treatment-, and health related instruments [14]. To our knowledge, this is the first study, which used a measure of interpersonal problems to validate both versions of the HAQ in a sample of persistently depressed patients. We found that patients’ ratings of the global helping alliance were significantly and negatively related to a sum score of interpersonal problems. This means that the more interpersonal problems a patient had before therapy, the more negative was the evaluation of the helping alliance. Additionally, we found that the more severe problems a patient had with being too dominant, too hostile or too hostile-submissive (i.e., subscales domineering/ controlling, vindictive/ self-centred, cold/ distant, socially inhibited), the more negative the patient perceived the global alliance. These results are in line with research that found that being too hostile [21] or too hostile-dominant [42] negatively impacts the helping alliance at treatment begin. What is more, our finding that patients who are too hostile-submissive evaluate the alliance with the therapist more negatively fits McCullough’s assumption that persistently depressed patients exhibit passive, submissive, and hostile behaviours towards the therapist which impedes the interaction with the therapist [23].

Looking at the subscales of the HAQ-P, the results are generally similar: the higher the interpersonal distress and the more problems a patient has with being too hostile-dominant, hostile and hostile-submissive, the more negative the patient evaluated the ‘satisfaction with therapeutic outcome’. Therapists’ evaluation of the global alliance however, was significantly related only to patients’ interpersonal problems with being too dominant or too hostile-submissive.

These findings are principally in line with our expectations. Yet, just part of our hypotheses was confirmed: Only the patient rated global helping alliance correlated consistently with patients’ pre-treatment problems with being too dominant, hostile-dominant, hostile, hostile-submissive, and the total IIP score. For the subscales and the therapist-rated alliance the correlational matrix was not so consistent. Moreover, contrary to our hypothesis, we did not find any (positive) correlations between the HAQ and interpersonal problems relating to being overly friendly-submissive.

One must acknowledge that the magnitude of the association between facets of the IIP and the HAQ is only weak [43]. At the same time, the association is comparable in size to a study by Puschner et al. [21]. Therefore, while the results are relevant to the important question of how interpersonal problems are related to the helping alliance, the IIP may not be the most suitable instrument to establish external validity of the HAQ. This is because one may not expect medium or high correlations. Having said that, correlation coefficients in our study may reflect the homogeneity of our sample (persistent depressive patients), which may have decreased the variance in our data.

Our findings must be viewed considering some limitation the study holds. Firstly, we did not check for a socially desirable response stile, i.e. evaluating the alliance more positive than actually perceived, of neither patients nor therapists. In addition to that, not taking into account the hierarchical structure of the data (i.e., several patients were treated by the same therapist) is a drawback: Theoretically, it is possible that the alliance construct is unidimensional on one level, but two-dimensional on the other level. Differences between our results and those in other studies may also be accounted for by the hierarchical structure. Yet, the relatively big sample size and the multi-centre approach of the study support the generalizability of the results. To the best of our knowledge, this was the first study to run confirmative factor analyses on both versions of the German HAQ and to employ a measure of patients’ pre-treatment interpersonal problems as a criterion for convergent validity.

## Conclusion

The HAQ is a reliable instrument. CFA did not clearly recommend a two-factorial model over a one-factorial model or vice versa. Thus, our findings suggest using the instrument uni- or twodimensionally, i.e. to work with the global alliance scale or the subscales ‘relation to the therapist/ patient’ and ‘satisfaction with therapeutic outcome’. Patients’ and therapists’ perception of the alliance is related to pre-treatment interpersonal problems of the patient. These findings are particularly relevant to research on and with the HAQ as it is a standard, perhaps most widely used instrument in current psychotherapy research [1, 16], especially in German speaking countries [14]. Moreover, our results contribute to the ongoing debate on the factor structure of the HAQ (e.g., [14, 20]).

In summary, the HAQ, both for patients and therapists, is an economically applicable research instrument. It assesses the therapeutic alliance via two subscales ‘relation to the therapist/ patient’ and ‘satisfaction with therapeutic outcome’ or one global scale. Moreover, its common usage in previous research, its brevity, the option to use it one- or two dimensionally, and its ability to measure changes in alliance over time [17] make the HAQ a recommendable instrument.

## Abbreviations

CBASP: Cognitive Behavioral Analysis System of Psychotherapy
CFI: Comparative Fit Index
CTQ: Childhood Trauma Questionnaire
df: Degrees of Freedom
DSM-IV: Diagnostic and Statistical Manual of Mental Disorders, fourth edition
HA1: Helping Alliance dimension 1
HA2: Helping Alliance dimension 2
HAQ: Helping Alliance Questionnaire
HAq-II: revised version of the HAQ
HAQ-P: Helping Alliance Questionnaire for patients
HAQ-T: Helping Alliance Questionnaire for therapists
HRSD: Hamilton Rating Scale for Depression
IIP: Inventory of Interpersonal Problems
M: Mean
MDD: Major Depressive Disorder
RCT: Randomized Controlled Trial
RMSEA: Root Mean Square Error of Approximation
SCID: Structured Clinical Interview for DSM-IV
SD: Standard Deviation
SP: Supportive Psychotherapy
SRMR: Standardized Root Mean Square Residual
TLI: Tucker Lewis Index
